# Basal cell carcinomas in organ transplant recipients versus the general population: clinicopathologic study

**DOI:** 10.1007/s00403-022-02403-6

**Published:** 2022-10-25

**Authors:** Nirmala Pandeya, Nancy Huang, Zainab Jiyad, Elsemieke I. Plasmeijer, Mandy Way, Nicole Isbel, Scott Campbell, Daniel C. Chambers, Peter Hopkins, H. Peter Soyer, David C. Whiteman, Catherine M. Olsen, Adele C. Green

**Affiliations:** 1 Population Health Department, QIMR Berghofer Medical Research Institute, Brisbane, Australia; 2 Department of Dermatology, St George’s Hospital, London, UK; 3 The Netherlands Cancer Institute, Amsterdam, The Netherlands; 4 Department of Nephrology, University of Queensland at Princess Alexandra Hospital, Brisbane, Australia; 5 Queensland Lung Transplant Service, The Prince Charles Hospital, Brisbane, Australia; 6 School of Clinical Medicine, The University of Queensland, Brisbane, QLD Australia; 7 Dermatology Research Centre, The University of Queensland Diamantina Institute, The University of Queensland, Brisbane, QLD Australia; 8 Department of Dermatology, Princess Alexandra Hospital, Brisbane, QLD Australia; 9 CRUK Manchester Institute and Faculty of Biology, Medicine and Health, University of Manchester, Manchester Academic Health Sciences Centre, Manchester, UK

**Keywords:** Skin cancer, Organ transplant recipients, Basal cell carcinoma, Immunosuppression, Histopathology

## Abstract

Organ transplant recipients (OTRs) are at greater risk of basal cell carcinomas (BCCs) than non-OTRs, but histopathologic differences between BCCs in OTRs and the general population are largely unknown. We compared clinicopathologic features of BCCs in OTRs vs the general population in Queensland, Australia. Details of BCC tumors (site, size, level of invasion, subtype, biopsy procedure) were collected from histopathology reports in two prospective skin cancer studies, one in OTRs and one general-population-based. We used log-binomial regression models to estimate age- and sex-adjusted prevalence ratios (PR) with 95% confidence intervals (CIs) for BCC features. Overall, there were 702 BCCs in 200 OTRs and 1725 BCCs in 804 population cases. Of these, 327 tumors in 128 OTRs were higher risk BCCs (any head and neck BCC; ≥ 2 cm on trunk/extremities), more per person than 703 higher risk BCCs in 457 cases in the general population (chi-square *p* = 0.008). Among head/neck BCCs, OTRs were more likely than general population cases to have BCCs on scalp/ear than on face/lip/neck (PR = 1.5, 95%CI 1.2–1.8). Although aggressive subtypes were less common among higher risk BCCs in OTRs, BCCs invading beyond the dermis were almost twice as prevalent in OTRs (PR = 1.8, 95% CI 1.3–2.6) than the general population.

**Supplementary Information:**

The online version contains supplementary material available at 10.1007/s00403-022-02403-6.

## Introduction

Organ transplant recipients (OTRs) are at greater risk of developing skin cancer due to their immunosuppressive medication regimens than non-OTRs [1]. The most frequently occurring skin cancers in OTRs are squamous cell carcinomas (SCCs) [2] and because they are responsible for high morbidity and mortality, SCCs in OTRs have been studied extensively [3]. Basal cell carcinomas (BCCs) occur less frequently than SCCs and rarely metastasise, and thus BCCs in OTRs have received relatively little attention despite their increased incidence compared with the general population [1, 2, 4]. Evidence shows that apart from immunosuppression, the personal and clinical factors associated with BCC in OTRs are similar to those in the general populations [4–6]. The mechanisms underlying their increased occurrence in OTRs are not well understood [7].

Insights may come from detailed investigation of BCC histopathology in OTRs, yet only four previous studies have directly compared the features of BCC tumors in OTRs versus non-immunosuppressed cases referred for hospital treatment. The earliest comparative study was conducted in a Dutch tertiary hospital where all BCCs diagnosed in the dermatology and plastic surgery departments 1985–1995 were reviewed. They found an increased percentage of superficial BCCs in OTRs (25%: 34 of 136) compared with other patients (16%: 452 of 2854) and BCCs in OTRs were more often on the trunk (39% vs 21%) and arms (8% vs 3%) [8]. In a second study in a French hospital over a 20-year period, 176 BCCs diagnosed in OTRs were identified and compared with 153 BCCs in immunocompetent patients randomly selected from records [9]. Compared with BCCs in other patients, BCCs in OTRs occurred on sites other than the head and neck (38% in OTRs vs 25%), and again comprised more superficial BCCs (34% in OTRs vs 14%) [9]. A third study in a tertiary hospital in London, UK, compared 100 consecutive cases of BCCs in non-immunosuppressed patients with 125 consecutive primary BCCs in renal transplant recipients diagnosed 1995–1997 [10]. Like the French study, the UK study found transplant tumors occurred more often on sites other than the head and neck (35% vs 19%), were more frequently of superficial subtype (29% vs 14%) and less often of micronodular (12% vs 24%) or infiltrative (15% vs 23%) subtypes than BCCs in non-transplant patients [10]. The most recent study by Krynitz et al. [11] in Sweden is the only one to date to compare BCCs (*n* = 341) in OTRs vs BCCs (*n* = 289,498) in the general population (as opposed to selected cases in tertiary treatment centers). Although this Swedish study’s main aim was to compare risk of developing BCC in OTRs (transplanted 2004–2011) and the general population, they also compared the anatomical site and histological type of BCCs in each group. They found no major differences, apart from superficial BCCs occurring more frequently in OTRs (20%) than the population at large (10%) [11]. Against this background, we aimed to compare histopathology features in two large BCC case series in Queensland, Australia, drawn from OTRs and the general population respectively.

## Methods

Similar data were obtained from two prospective skin cancer studies: the Skin Tumors in Allograft Recipients (STAR) study of OTRs, and the QSkin Sun and Health Study (QSkin) conducted in the general population. Details of the respective study designs and data collection methods have been published previously [4, 12, 13]. Both studies were approved by institutional ethics committees. In brief, participants in the STAR study were OTRs transplanted a year or more, recruited from the two transplant centers for Queensland. They comprised population-based lung transplant recipients, and kidney and liver transplant recipients deemed at high skin cancer risk because of a history of skin cancer or actinic keratosis, or else aged > 40 years, or immunosuppressed > 10 years. At baseline in 2012, OTRs provided demographic, phenotypic, sun exposure and other health-related information and received a full body skin examination by dermatology-trained doctors. Patients were followed up for new skin cancers until 2016 by annual dermatologic examinations, and interim skin cancers were notified by self-report and confirmed against histological records [4, 12]. All STAR study participants with at least one histologically diagnosed BCC were included in the present study.

QSkin is a large population-based cohort study of skin cancer with outcome data available for the follow-up period 2010–2014. Participants were recruited from Queensland’s compulsory electoral register with a 23% participation rate [13]. They provided demographic, phenotypic, sun exposure and other health-related information at baseline (2010) and consented to Medicare (Australian universal health insurance provider for care outside public hospitals) data linkage for ascertainment of skin cancer treatments (which mostly occur outside public hospitals) which were in turn linked with histopathology records. A random third of QSkin participants (selected by computer-generated random numbers) with at least one histopathologically diagnosed BCC during study follow-up (Jan 2012–Jun 2014) were included in this study.

### Outcomes

Pathology reports of all BCCs were reviewed by trained clinicians and details of body site and biopsy type (excision; punch/shave/curettage—collectively termed ‘partial biopsies’), whether information was given about biopsy margins (yes, no) and if yes, if margins were clear of tumor or not clear (involved tumor), depth of invasion (dermis, fat, muscle), and details of tumor subtype [morphoeic/sclerosing, infiltrative, micronodular, basosquamous—all classified as higher risk [14] (here ‘aggressive’); nodular, superficial—classified as lower risk [14] (‘non-aggressive’)] were extracted using a standard template. When a single BCC had more than one biopsy and histopathology report (e.g. diagnostic punch biopsy followed by excision), histopathology details were extracted from the most detailed report and supplemented by extra information from subsequent reports as applicable. BCCs of more than one subtype were classified based on the most aggressive subtype present.

### Statistical analysis

Frequency distributions of BCC characteristics in OTRs and the general population were compared using Chi-square distributions. We used a log binomial regression model with a binary outcome indicator for OTRs vs general population BCCs and estimated the prevalence ratio (PR) adjusting for age at baseline and sex. We used generalised estimating equations (GEE) approach in the regression model to adjust for the intra-individual correlations among multiple BCC lesions diagnosed in the same individual.

## Results

There were 702 BCCs from 200 OTRs with at least 1 histopathologically diagnosed BCC in the STAR study, together with 1725 BCCs from the 804 randomly selected QSkin cases from the general population (Table 1). The OTR cases comprised more men (76%) than general population cases (56%) but there was no difference in baseline age between the two groups. The proportion of OTR cases who had their skin checked more than once a year was 53%, compared with 21% of BCC cases from the general population.

**Table 1:** Clinical characteristics of BCC tumors in organ transplant recipients and in randomly selected general population cases stratified by diagnostic procedure

	BCCs in organ transplant recipients (*N* = 702)		BCCs in general population (*N* = 1725)^a^	
	Excision *n* (%)	Punch/shave biopsy or curettage *n* (%)	Excision *n* (%)	Punch/shave biopsy or curettage *n* (%)
Overall	535	167	1111	599
Body site				
Head/neck	248 (46.4)	70 (41.9)	482 (43.4)	200 (33.4)
Scalp	16 (6.5)	2 (2.9)	17 (3.5)	5 (2.5)
Ear	45 (18.2)	17 (24.3)	50 (10.4)	14 (7.0)
Face	152 (61.3)	39 (55.7)	303 (62.9)	143 (71.5)
Lip	5 (2.0)	6 (8.6)	21 (4.4)	8 (7.0)
Neck	30 (12.1)	6 (8.6)	91 (18.9)	30 (15.0)
Arms/hands	98 (18.3)	23 (13.8)	169 (15.2)	81 (13.5)
Trunk	122 (22.8)	50 (29.9)	346 (31.1)	231 (38.6)
Legs/feet	67 (12.5)	23 (13.8)	112 (10.1)	86 (14.4)
Missing	–	1 (0.6)	2 (0.18)	1 (0.17)
Tumor size				
< 2.0 cm	459 (85.8)	41 (24.6)	1005 (90.5)	197 (32.9)
2 cm or larger	18 (3.4)	–	19 (1.7)	–
Missing	58 (10.8)	126 (75.5)	87 (7.8)	402 (67.1)
Tumor depth (minimum)				
Dermis	331 (61.9)	28 (16.8)	936 (84.3)	165 (27.6)
Fat	12 (2.2)	2 (1.2)	14 (1.3)	–
Muscle	1 (0.2)	–	3 (0.3)	–
Unknown	191 (35.7)	137 (82.0)	158 (14.2)	434 (72.5)
Tumor type^b^				
Non-aggressive	436 (82.0)	133 (83.1)	759 (68.6)	484 (83.2)
Aggressive	96 (18.1)	27 (16.9)	347 (31.4)	98 (16.8)

^a^15 people had diagnostic procedure missing

^b^Non-aggressive subtypes: nodular, superficial; aggressive subtypes: morphoeic, infiltrative, micronodular, basosquamous

Substantial proportions of BCC tumors in each group (*n* = 167, 24% OTRs; *n* = 599, 35% general population) were diagnosed and treated only by partial biopsies, thus tumor size and depth of invasion were unknown for over two-thirds of partially excised BCCs in both case groups (Table 1). Furthermore, two-thirds of BCCs diagnosed by biopsies in OTRs and one-third in the general population had no statement in the histopathology report about tumor margin involvement (Supp Table 1), though this reflected the case that most of the BCCs biopsied rather than excised were small BCCs occurring on the trunk or extremities at low risk of recurrence and mortality [15, 16]. Subsequent analyses therefore focused on higher risk BCCs, defined as in previous reports as any BCCs on the head and neck, and BCCs measuring ≥ 2 cm on the trunk or extremities [15, 16].

There were 327 higher risk BCCs in 128 OTRs, more per person than in the general population with 703 in 457 cases (chi-square *p* = 0.008). Excision was the definitive diagnostic procedure for 257 (79%) of higher risk BCCs in OTRs and 496 (71%) of higher risk BCCs in the general population. In line with our definition of higher risk BCCs, almost all were on the head and neck (97% OTRs; 98% general population) (Table 2). Head and neck BCCs were more prevalent on the scalp or ear (80, 25%) in OTRs than in the general population (86, 13%) (*p* < 0.001).

**Table 2:** Clinical characteristics of high-risk^a^ BCC tumors in organ transplant recipients and in randomly selected general population cases stratified by diagnostic procedure

	BCCs in organ transplant recipients (*N* = 327)		BCCs in general population (*N* = 696)	
	Excision *n* (%)	Punch/shave biopsy or curettage *n* (%)	Excision *n* (%)	Punch/shave biopsy or curettage *n* (%)
Overall	257	70	496	200
Body site				
Head/neck	248 (96.5)	70 (100)	482 (97.2)	200 (100)
Scalp	16 (6.5)	2 (2.9)	17 (3.5)	5 (2.5)
Ear	45 (18.2)	17 (24.3)	50 (10.4)	14 (7.0)
Face	152 (61.3)	39 (55.7)	303 (62.9)	143 (71.5)
Lip	5 (2.0)	6 (8.6)	21 (4.4)	8 (7.0)
Neck	30 (12.1)	6 (8.6)	91 (18.9)	30 (15.0)
Arms/hands	3 (1.2)	–	5 (1.1)	–
Trunk	4 (1.6)	–	4 (0.8)	–
Legs/Feet	2 (0.8)	–	5 (1.0)	–
Tumor size				
< 2.0 cm	211 (82.1)	24 (34.3)	429 (86.5)	54 (27)
2 cm or larger	18 (7.0)	–	19 (3.8)	–
Missing	28 (10.9)	46 (65.7)	48 (9.7)	146 (73)
Tumor depth				
Dermis	167 (65.0)	13 (18.6)	403 (81.1)	40 (20.0)
Fat	12 (4.7)	1 (1.4)	9 (1.8)	–
Muscle	1 (0.4)	–	3 (0.6)	–
Unknown	77 (30.0)	56 (80.0)	81 (16.3)	160 (80.0)
Tumor type^b^				
Non-aggressive	191 (74.9)	50 (76.9)	285 (57.8)	140 (72.2)
Aggressive	64 (25.1)	15 (23.1)	208 (42.2)	54 (27.8)

^a^High-risk skin tumors were defined as BCC tumors occurring on the head and neck or having a tumor size of ≥ 2 cm if on other body sites

^b^Non-aggressive subtypes: nodular, superficial; aggressive subtypes: morphoeic, infiltrative, micronodular, basosquamous

Of higher risk BCCs that were excised, 7% (18) in OTRs were ≥ 2 cm in diameter compared with 4% (19) in the general population (*p* = 0.05), and 5% (13) in OTRs were invading beyond the dermis compared with 2% (12) in the general population (*p* = 0.06) (Table 2). All higher risk BCCs diagnosed by partial biopsy that had available data were < 2 cm in diameter and invasion was restricted to the dermis with the exception of one BCC in an OTR invading subcutaneous fat (Table 2). Margins were reportedly involved in 11% (29) excised higher risk BCCs in OTRs and 12% (61) in the general population (Supp Table 2).

With respect to tumor subtype, around 24% of higher risk BCCs in OTRs were classified as aggressive subtypes compared with 38% in the general population (Table 2). Overall, the lower percentage with aggressive subtypes in OTRs vs the general population reflected OTRs’ smaller proportions of infiltrative (6% vs 14%) and micronodular (1% vs 14%) subtypes, and proportionately more superficial BCCs in OTRs (34% vs 21%) (Suppl table 2).

After adjusting for age and sex (and excluding BCCs with missing information), BCCs on scalp or ear (vs other head and neck subsites) were 1.5 times more prevalent in OTRs than in the general population (PR = 1.5, 95% CI 1.2–1.8). Similarly, the prevalence of higher risk BCCs measuring ≥ 2 cm (versus < 2 cm) was 1.3 times greater in OTRs (PR = 1.3, 95% CI 0.9–1.8), while prevalence of BCCs invading deeper than the dermis was 1.8 times greater in OTRs (PR = 1.8, 95% CI 1.3–2.6). On the other hand, the prevalence of aggressive vs nonaggressive subtypes in OTRs was lower than in the general population (PR = 0.7, 95% CI 0.5–0.9) (Table 3).

**Table 3:** Prevalence ratios by clinical characteristics of high-risk BCCs and all BCCs in organ transplant recipients compared with general population cases

	High-risk BCCs	All BCCs
	Age- and sex-adjusted PR (95% CI)	Age- and sex-adjusted PR (95% CI)
Head and neck site		
Face, lip and neck	Reference	Reference
Scalp or ear	1.5 (1.2–1.8)	2.0 (1.3–2.9)
Tumor size		
< 2 cm	Reference	Reference
2 cm or larger	1.3 (0.9–1.8)	1.4 (0.9–2.0)
Tumor depth (minimum level of invasion)		
Dermis	Reference	Reference
Deeper than dermis	1.8 (1.3–2.6)	1.9 (1.3–2.8)
BCC subtype^a^		
Non-aggressive	Reference	Reference
Aggressive	0.7 (0.5–0.9)	0.7 (0.6–0.9)

^a^Non-aggressive subtypes: nodular, superficial; aggressive subtypes: morphoeic, infiltrative, micronodular, basosquamous

When we repeated these analyses to include low-risk BCCs, estimated PRs were very similar to those for higher risk BCCs only (Table 3).

## Discussion

We compared the clinicopathologic details of BCCs diagnosed in OTRs vs BCCs in the general population, and found that a substantial proportion of BCCs (24% and 35% in these groups, respectively) were diagnosed and treated using only punch or shave biopsies or curettage. This severely limited the histopathological comparison of all study BCCs, because neither their size nor depth of invasion, the major prognostic markers, could be properly assessed. These key prognostic data were not available in two-thirds of BCCs diagnosed by punch/shave biopsies or curettage in OTRs and one-third of general population cases. We, therefore, restricted further analyses to higher risk BCCs that carry the worst prognosis [15, 16] and were more often treated with excision. Higher risk BCCs comprised a higher proportion (47%) of all study BCCs in OTRs than in the general population (40%) suggesting at the outset a worse prognosis of BCCs in OTRs.

Among higher risk BCCs, OTRs scored worse on all major prognostic indicators compared with the general population. By definition almost all higher risk BCCs were on the head and neck, but in OTRs head and neck BCCs were 50% more prevalent on the scalp or ear (subsites at high risk of spread) compared with BCCs in the general population after adjusting for age and sex. The same increase of BCCs on the ears of OTRs was seen in the French series overall (despite fewer BCCs on the head and neck overall compared with immunocompetent French cases) [9]. In addition, prevalence of BCCs invading beyond the dermis were almost twice as common in OTRs than in the general population. Paradoxically, there was a 30% reduced likelihood of BCCs of aggressive histological subtypes diagnosed in OTRs than in the general population, and this is consistent with findings of all four previous studies which observed the same excess of non-aggressive BCC subtypes in OTRs, specifically the same excess of superficial BCCs [8–11]. This universal finding may be influenced to some extent by the recommended destructive treatment of superficial BCCs [17] that often occurs in the general population without histology [18], thus such superficial BCCs will not be captured in histopathologic studies.

This is among the largest clinicopathological series of BCCs in OTRs reported to date, and one of the few to compare BCCs in OTRs and the general population. In contrast, SCCs have been more widely studied in OTRs, because the risk is substantially higher [11, 19], and their outcomes have also been shown to be poorer than in the population at large, with higher mortality [1, 3]. The prospective design allowed us to focus on newly developed BCCs in the OTRs in particular, because they had annual dermatologic examinations, so that histologic features of BCC diameter and depth of invasion were not potentially magnified by delay in diagnosis. Thus, our comparisons were conservative. There was a large amount of unavailable information on tumor size and depth which could be partly traced to high proportions of BCCs overall that were diagnosed and treated with punch/shave biopsies or curettage. We thus focused on higher risk BCCs as others have done [15], as these are more often excised and are of greatest concern clinically [15, 16]. All else being equal, the clinical course of a BCC tumor is an indication of its aggressiveness, but since we did not have information on the clinical history, management or outcomes of BCCs in this study, we used tumor features (≥ 2 cm; head and neck location) on trunk/extremities shown elsewhere to be associated with BCC metastasis/death [15] as indicators of clinical outcome. We relied on these factors as reported by the original diagnosing histopathologist, as it was not feasible to organise central review of the over 2000 BCCs in this study. We also note that in the final analysis, we found that prevalence ratios were hardly different when we considered all BCCs or only higher risk BCCs.

In conclusion, our data provide support for the notion that systemic immunosuppression promotes not only the development of BCC [11, 19] (albeit to a lesser extent than for SCC), but also the progression of BCC, with a greater prevalence of deep invasion in OTRs, including in those completely excised. Patient care and future research to confirm these findings may both benefit from enhanced communication between treating clinicians and dermatopathologists, not only via the pathology requisition [20] but also by comprehensive histopathologic reporting of BCCs.

## Supplementary Information

Below is the link to the electronic supplementary material.

Supplementary file1 (DOCX 29 KB)
