# Supplementary material for: Basal cell carcinomas in organ transplant recipients versus the general population: clinicopathologic study
Source: Arch Dermatol Res. 2022 Oct 25;315(4):771–7. doi: 10.1007/s00403-022-02403-6 (PMC10085887; doi:10.1007/s00403-022-02403-6)
Supplement: Supplementary file 1 — Supplementary file1 (DOCX 29 KB) [file 403_2022_2403_MOESM1_ESM.docx]

**Supplementary table 1. Margin involvement and tumor subtypes of BCCs in organ transplant recipients and in general population cases stratified by diagnostic procedure**

|  | **Organ transplant recipients**  **(n=702)** | | **General population**  **(n=1725)** | |
| --- | --- | --- | --- | --- |
|  | **Excision** | **Punch/shave biopsy or**  **curettage** | **Excision** | **Punch/shave biopsy or**  **curettage** |
| **Margin involvement** |  |  |  |  |
| Margin Clear | 492 (92.0) | 13 (7.8) | 976 (87.9) | 78 (13.0) |
| Margin involved | 39 (7.3) | 40 (24.0) | 104 (9.4) | 313 (52.3) |
| Not stated | 4 (0.8) | 114 (68.3) | 31 (2.8) | 208 (34.7) |
| **Tumor subtype** |  |  |  |  |
| Superficial | 239 (44.7) | 82 (49.1) | 385 (34.7) | 295 (49.3) |
| Nodular | 197 (36.8) | 51 (30.5) | 374 (33.7) | 189 (31.6) |
| Infiltrative | 30 (5.6) | 4 (8.4) | 133 (12.0) | 36 (6.0) |
| Morphoeic/Sclerosing | 50 (9.4) | 14 (8.4) | 61 (5.5) | 19 (3.2) |
| Basosquamous | 10 (1.9) | 7 (4.2) | 26 (2.3) | 4 (0.7) |
| Micronodular | 6 (1.1) | 2 (1.2) | 127 (11.4) | 38 (6.3) |
| Missing | 3 (0.6) | 7 (4.2) | 5 (0.5) | 18 (3.0) |

**Supplementary table 2. Margin involvement and tumor subtypes of high-risk BCCs in organ transplant recipients and in general population cases stratified by diagnostic procedure**

|  | **Organ transplant recipients (N=327)** | | **General population (N=696)** | |
| --- | --- | --- | --- | --- |
|  | **Excision** | **Punch/shave biopsy or**  **curettage** | **Excision** | **Punch/shave biopsy or**  **curettage** |
| **Margin involvement** |  |  |  |  |
| Margin Clear | 226 (87.9) | 3 (4.3) | 425 (85.7) | 18 (9.0) |
| Margin involved | 29 (11.3) | 18 (25.7) | 61 (12.3) | 120 (60.0) |
| Not stated | 2 (0.8) | 49 (70.0) | 10 (2.0) | 62 (31.0) |
| **Tumor subtype** |  |  |  |  |
| Superficial | 91 (35.4) | 21 (30.0) | 91 (18.4) | 54 (27.0) |
| Nodular | 100 (38.9) | 29 (41.4) | 194 (39.1) | 86 (43.0) |
| Infiltrative | 17 (6.6) | 4 (5.7) | 80 (16.1) | 16 (8.0) |
| Morphoeic/Sclerosing | 36 (14.0) | 8 (11.4) | 40 (8.1) | 13 (6.5) |
| Basosquamous | 7 (2.7) | 3 4.3) | 13 (2.6) | 2 (1.0) |
| Micronodular | 4 (1.6) | - | 75 (15.1) | 23 (11.5) |
| Missing | 2 (0.8) | 5 (7.1) | 3 (0.6) | 6 (3.0) |
